# Genetic purging in captive endangered ungulates with extremely low effective population sizes

**DOI:** 10.1038/s41437-021-00473-2

**Published:** 2021-09-28

**Authors:** Eugenio López-Cortegano, Eulalia Moreno, Aurora García-Dorado

**Affiliations:** 1 Institute of Evolutionary Biology, University of Edinburgh, EH9 3FL Edinburgh, UK; 2 Estación Experimental de Zonas Áridas (CSIC), 04120 Almería, Spain; 3 Universidad Complutense, 28040 Madrid, Spain

**Keywords:** Population genetics, Inbreeding, Conservation biology

## Abstract

Inbreeding threatens the survival of small populations by producing inbreeding depression, but also exposes recessive deleterious effects in homozygosis allowing for genetic purging. Using inbreeding-purging theory, we analyze early survival in four pedigreed captive breeding programs of endangered ungulates where population growth was prioritized so that most adult females were allowed to contribute offspring according to their fitness. We find evidence that purging can substantially reduce inbreeding depression in *Gazella cuvieri* (with effective population size *N*_*e*_ = 14) and *Nanger dama* (*N*_*e*_ = 11). No purging is detected in *Ammotragus lervia* (*N*_*e*_ = 4), in agreement with the notion that drift overcomes purging under fast inbreeding, nor in *G. dorcas* (*N*_*e*_ = 39) where, due to the larger population size, purging is slower and detection is expected to require more generations. Thus, although smaller populations are always expected to show smaller fitness (as well as less adaptive potential) than larger ones due to higher homozygosis and deleterious fixation, our results show that a substantial fraction of their inbreeding load and inbreeding depression can be purged when breeding contributions are governed by natural selection. Since management strategies intended to maximize the ratio from the effective to the actual population size tend to reduce purging, the search for a compromise between these strategies and purging could be beneficial in the long term. This could be achieved either by allowing some level of random mating and some role of natural selection in determining breeding contributions, or by undertaking reintroductions into the wild at the earliest opportunity.

## Introduction

The decline in fitness due to inbreeding is one of the major threats menacing the survival of small, endangered populations (Lande 1994; Hedrick and Kalinowski 2000; Crow 2008; Frankham 2010; Frankham et al. 2014a). Large populations harbor many (partially) recessive deleterious mutations segregating at low frequency whose effects hide in heterozygosis but, under inbreeding, increase their expression in homozygosis (Charlesworth and Willis 2009). This exposure of recessive effects produces inbreeding depression but also causes an enhancement of natural selection that has been referred to as genetic purging (Crow 1970; Hedrick 1994) and that can substantially reduce both inbreeding depression and inbreeding load (Wang and Hill 1999; García-Dorado 2012; Charlesworth 2018).

Genetic purging, however, has received less attention than inbreeding depression in the literature (Keller and Waller 2002; Bouzat 2010; Hedrick and García-Dorado 2016) as it is more difficult to detect. This is partly because it is expected to be more successful but also slower under slower inbreeding, so that a large number of generations may be required before the effects of purging become apparent (García-Dorado 2012; López-Cortegano et al. 2018). This may explain why purging has been mainly detected in experimental populations of the fruit fly *Drosophila melanogaster* (Latter et al. 1995; Swindell and Bouzat 2006a; Ávila et al. 2010; Pekkala et al. 2012; Bersabé and García-Dorado 2013, López-Cortegano et al. 2016). However, purging has rarely been documented in nature (Byers and Waller 1999; Crnokrak and Barrett 2002) and many studies aimed to detect genetic purging have failed or found inconsistent results among species, both in captive and wild populations (Ballou 1997; Boakes et al. 2007; Leberg and Firmin 2008; Kennedy et al. 2014).

Genetic purging is relevant to biodiversity conservation. For example, it can be critical to determine the minimum size (MVP) proposed for a population to survive in situ in the short-medium term (García-Dorado 2015; Caballero et al. 2017).

Regarding ex situ conservation, there is consensus in that inducing purging through intentional inbreeding implies unacceptable risks for critically endangered populations (Frankham et al. 2014a; Hedrick and García-Dorado 2016; de Cara et al. 2013). However, it is not clear to what extent unintentional inbreeding due to the reduced population size can be tolerated because of purging. Therefore, there could be situations where allowing some level of random mating and letting breeding contributions be determined by natural selection could be beneficial in the long term. Furthermore, while many estimates of the inbreeding load have been given for both captive and wild populations (Ralls et al. 1988; O’Grady et al. 2006), so far estimates of the genetic parameter that determines the consequences of genetic purging (see below) have only been obtained in experimental conditions where purging has been found to be relevant, particularly under competitive conditions (Bersabé and García-Dorado 2013; López-Cortegano et al. 2016).

Here we analyze inbreeding depression and purging in four captive populations belonging to different threatened ungulate species of the Family Bovidae with different demographic histories: barbary sheep (*Ammotragus lervia*), Cuvier’s gazelle (*Gazella cuvieri*), dorcas gazelle (*G. dorcas*), and dama gazelle (*Nanger dama*). For all these populations, inbreeding depression has been detected in the past regarding fitness traits such as longevity, reproductive success, survival, or parasite burden (Cassinello and Alados 1996; Cassinello et al. 2001; Cassinello 2005). However, more recent studies on Cuvier’s and dama gazelles have suggested that purging might be actively reducing the harmful consequences of inbreeding (Ibáñez et al. 2011, 2014; Moreno et al. 2015). Our results show evidence of purging for early survival in these two species. No significant purging was estimated for *A. lervia*, where fewer data were available, nor for *G. dorcas*, where the number of generations available was very likely too small for purging to be detected. This study illustrates how the consequences of inbreeding and purging upon fitness traits can be analyzed in ex situ conservation programs, as well as the potential implications for the management of endangered species.

## Methods

### Study species and captive populations

The four species studied are threatened ungulates listed in the International Union for Conservation of Nature (IUCN) Red List (*N. dama* as “critically endangered,” and the others as “vulnerable”) (IUCN 2019). Their populations have steeply declined in the wild since the 1950s, apparently due to excessive hunting and habitat degradation in their range in Northern Africa. In the 1970s, a few individuals of *A. lervia* (spp. *sahariensis), G. cuvieri*, *G. dorcas (*spp. *neglecta*), and *N. dama (*spp. *mhorr)* arrived at “La Hoya” Experimental Field Station (EEZA-CSIC; Almería, Spain; Cano 1988), and captive populations were established. All these ungulates are polygynous.

At La Hoya, breeding groups comprised one male and a group of 5–8 females and were designed to minimize coancestry between mating individuals, breeding males being chosen on the basis of their minimum coancestry with the females in their harem. The number of breeding offspring contributed per female was not determined by the management protocol. In the case of *G. cuvieri* and *N. dama*, starting from year 2000 males were replaced every autumn in order to increase the number of males with breeding opportunities. Some details for the biology of each species are given in Appendix S1. Extensive information can be found in Beudels et al. 2005 (for the gazelle species) and Cassinello 1998 (for barbary sheep).

### Pedigree records processing and traits analyzed

Since captive populations were first established, records have been kept including dates of birth and death, dam and sire identities, and the sexes of all individuals (records freely available at “La Hoya” Experimental Field Station institutional website as international studbooks: http://www.eeza.csic.es/es/programadecria.aspx, accessed in February 2020). Animals born in the wild were considered as unrelated founders. Other individuals with unknown or uncertain ancestry were removed from these pedigrees, as well as all their descendants, as their inbreeding and the purging accumulated in their descendants would be underscored.

As fitness traits, we considered early-life survival (survival here onwards: a dichotomous variable for surviving 15 days or not, *W*_*S*_) following Ibáñez et al. (2014), as well as lifetime female productivity measured as the total number of offspring born per female (*W*_*P*_). Note that, while most females are given free opportunities to breed, breeding males are chosen according to management criteria so that the number of offspring of each male is not a good measure of its genetic value for productivity. Table 1 provides a list with the terminology used for these traits and for the main parameters used. In both instances, the trait value was assumed to be unknown for founder individuals as they do not represent a random sample of the wild population, but a sample of those that survived and had offspring. Although the program supplied individuals to other institutions, only fitness data for individuals belonging to La Hoya (coded as “Almeria” location in the studbooks) were considered to prevent noise from environmental sources, as this population is the one with more individuals for all species and with a higher degree of pedigree completeness (see Table 2). *W*_*s*_ was not measured for individuals that died accidentally before 15 days old. As early deaths were always recorded, individuals lacking date of death were assumed to survive up to 15 days (*W*_*S*_ = 1), but their *W*_*P*_ (when females) was not measured. Similarly, females born so recently that they were still expected to breed by the end of the pedigree were excluded from the analysis of *W*_*P*_, as their true average reproductive fitness would be underscored. Therefore, when analyzing productivity, we did not consider the *W*_*P*_ values of females born in the last 16 years from the pedigree for *A. lervia*, 11 for *N. dama*, and 9 for *G. cuvieri* and *G. dorcas*. These values correspond to the rounded-down 90% percentile of longevity. The number of individuals filtered due to the above criteria is given in Appendix S2 and Supplementary Table S1.

**Table 1:** Main parameters used.

*W*	Fitness or a fitness trait: *W*_*S*_ is used for 15-day survival; *W*_*P*_ is used for overall female productivity
*δ*	Part of the genetic load that is hidden in heterozygosis. It gives the rate of fitness decline with increasing inbreeding that would be expected in the absence of selection. We use *δ* for the inbreeding load ascribed to the effects of the deleterious alleles in the genotype of the individual that is being assayed for the trait
*δ*_*M*_	Inbreeding load ascribed to the effects of the deleterious alleles in the genotype of the individual’s mother
*d*	Purging coefficient, representing the part of the deleterious effect that is exposed to genetic purging due to inbreeding
*F*	Wright’s inbreeding coefficient
*g*	Purged inbreeding coefficient, representing *F* adjusted by the deleterious frequency that is expected by considering purging
*S*	Effect on the trait of being a male
*POM*	Effect on the trait of the period of management with regular veterinary care
*YOB*	Effect of the year of birth
*W*_0_	Expected value for the trait in non-inbred individuals estimated from the inbreeding-purging model
*W’*_0_	Mean value of the trait computed in non-inbred individuals with no inbred ancestors (with its standard error)
*t*_*m*_	A value for the number of generations of inbreeding below which detect purging is unlikely
*N*_*e*_	Effective population size
*N*	Number of individuals in the pedigree
*EqG*	Number of equivalents complete generations, representing, for each individual, the number of generations in a complete pedigree that would account for its number of ancestors in the actual pedigree.
*TP*	The target population by the end of the pedigree: demographic parameters are estimated to account for the inbreeding of this TP
*AN*_*f*_	Actual number of founders in the pedigree
*N*_*f*_	Number of actual founders that are ancestors of the individuals of the TP
*N*_*ef*_	Effective number of founders of the target population, defined as the number of equally contributing founders that would account for the genetic diversity of the TP for inbreeding by descent.

**Table 2:** Summary population parameters: total number of individuals in the pedigree (*N*), pedigree completeness (%PC), mean number of equivalent complete generations in the target population *EqG* (±SE), number of individuals in the target population (*N*_*TP*_), effective population size *N*_*e*_ (±SE), actual number of founders (*AN*_*f*_) in the pedigree, number of founders of the TP (*N*_*f*_), and effective number of founders of the TP (*N*_*ef*_).

Species	*N*	%PC	*EqG*	*N*_*TP*_	*N*_*e*_	*AN*_*f*_	*N*_*f*_	*N*_*ef*_	*N*_*ef*_/*AN*_*f*_
*A. lervia*	380	99.5	5.81 (0.54)	80	3.83 (0.05)	3	2	1.77	0.59
*G. cuvieri*	948	99.5	8.77 (0.53)	176	14.01 (0.17)	5	4	3.58	0.72
*G. dorcas*	1279	95.9	7.13 (0.46)	283	39.32 (1.42)	37	20	13.39	0.36
*N. dama*	1316	99.6	8.13 (0.47)	251	11.10 (0.12)	5	4	2.61	0.52

For the four populations, analysis of these fitness traits included the individual sex (*S*), as mothers’ inbreeding differentially affects survival of sons and daughters for *G. cuvieri* (Moreno et al. 2015). Two environmental factors were also considered: year of birth (*YOB*) as numerical variable, and period of management (*POM*) as a categorical one that included two levels corresponding to the periods before and after the introduction of regular veterinary care (1993). The processed pedigree files can be downloaded from a GitLab repository (see Data availability statement below).

### Demographic analysis

To account for the existence of overlapping generations when estimating demographic parameters, we computed the number of equivalent complete generations (*EqG*_*i*_) for each individual *i* as the sum of (1/2)^*n*^ over all its known ancestors, where *n* is the number of generations that separate *i* from its ancestor (Boichard et al. 1997). *EqG*_*i*_ represents the number of generations in a complete pedigree that would account for the number of ancestors of individual *i*. Then, for each pedigree, we computed the effective population size over the whole captivity period from the rates of inbreeding estimated in a target population (TP) that consisted of the individuals with the largest *EqG* observed in the pedigree or one *EqG* less, verifying that both parents of each individual belonging to the TP were known. This TP roughly represents the individuals at the end of the pedigree. The realized effective population size (*N*_*e*_) was estimated as $N_e = \frac{1}{{2{\Delta}F}}$, where *ΔF* is the average of the estimates of the rate of inbreeding obtained for the individuals of the TP (${\Delta}F_i = 1 - \root {{EqG_i - 1}} \of {{1 - F_i}}$ for individual *i*), following the method developed by Gutiérrez et al. (2008, 2009).

Contributions of founders to the diversity of the TP regarding identity by descent were analyzed following methods in Tahmoorespur and Sheikhloo (2011). Thus, we computed: (i) the number of founders (*N*_*f*_), defined as ancestors of the TP with both parents unknown and (ii) the effective number of founders (*N*_*ef*_), defined as the number of equally contributing founders that would account for the genetic diversity of the TP regarding inbreeding by descent. Estimates of other related parameters can be found in Appendix S3.

### Inbreeding-purging analysis

We estimated the parameters that determine the evolution of fitness under the inbreeding-purging (IP) model outlined below (García-Dorado 2012), which deals with the inbreeding load ascribed to (partially) recessive deleterious alleles. Overdominance for fitness is not considered in this model. The expected fitness *W* (here survival *W*_*S*_ or productivity *W*_*P*_) of individual *i* is predicted as follows:

1 $$W_i = W_0{{{\rm{exp}}}}^{ - \delta g_i}$$

where *W*_0_ is the expected fitness of the reference non-inbred population that provided the founder individuals; *δ* is the rate of inbreeding depression of fitness that would be expected in the absence of selection, which is equivalent to the initial inbreeding load (often denoted by *B*) that can be interpreted as the haploid number of hidden lethal equivalents (Morton et al. 1956); and *g*_*i*_ is the purged inbreeding coefficient of individual *i*, which represents the expected value of *F*_*i*_ *q*_*i*_ */q*_*0*_, where *q*_*0*_ is the frequency of the deleterious allele in the base population that provided the founders of the pedigree, and *F*_*i*_ and *q*_*i*_ stand for Wright’s inbreeding coefficient and for the expected frequency of those deleterious alleles in individual *i*, respectively (García-Dorado 2012). This model provides good approximations for *dN*_*e*_ ≥ 1.

The purged inbreeding coefficient *g* depends on the purging coefficient *d* that, in a single locus model, is the recessive component of the deleterious effect that is expressed only in the homozygotes but is concealed in the heterozygotes [*d* = *s*(1/2–*h*), where *s* stands for the selection coefficient against the homozygote and *h* for the dominance coefficient]. The purging coefficient *d* takes values from 0 for additive gene action to 0.5 for a fully recessive lethal allele and determines both inbreeding depression and genetic purging. In a genome-wide multilocus approach, *d* represents an effective purging coefficient, i.e., the *d* value that, when used in the IP model, produces the best fit to the observed consequences of purging on fitness. Then, a small or non-significant *d* estimate can imply that the inbreeding load is mainly due to many alleles with small individual *d* values, or that inbreeding increases so fast (i.e., *N*_*e*_ is so small) that purging is overwhelmed by drift (*d* < 1/*N*_*e*_). On the contrary, a large *d* estimate means that a large fraction of the inbreeding load can be purged (*d* > 1/*N*_*e*_), and suggests that it is due to large individual *d* values. The expected purged inbreeding coefficient *g*_*i*_ can be computed either as a function of *N*_*e*_ or from the pedigree (García-Dorado 2012; García-Dorado et al. 2016).

The efficiency of purging can be defined as the proportional reduction it is expected to cause in the long term for the frequency of the deleterious alleles that accounted for the initial inbreeding load [i.e., by the expected (*q*_0_–*q*_*i*_)*/q*_0_]. To predict purging efficiency, we note that the *q*_*i*_*/q*_0_ ratio asymptotically expected with increasing generations can be predicted by the corresponding asymptotic purged inbreeding coefficient:

$$\widehat g = \frac{{1 - 2d}}{{1 + 2d\left( {2N - 1} \right)}}$$

Therefore, the efficiency of purging can be predicted as (1 – $\widehat g$), which increases with increasing *dN*_*e*_. For very large *dN*_*e*_, the deleterious alleles inherited from the base population that accounted for the initial inbreeding load are expected to be eventually removed by purging so that all the inbreeding depression they caused is finally reverted, but the process is very slow. As *d* approaches zero, the initial deleterious alleles are expected to be finally fixed or lost due to drift, and the efficiency of purging is zero (in this circumstance most natural selection is purging, but for expressions including non-purging selection see the Full Model in García-Dorado 2012). For a single locus, this measure of purging efficiency is approximately equal to the expected reduction of the fitness asymptotic inbreeding depression caused by purging.

Thus, purging under panmixia is never expected to improve fitness average above the value of the original panmictic population, so that a smaller population size is always worse than a larger one regarding fitness average. The reason is that, even if purging can reduce the overall burden of deleterious alleles during very long periods, those that persist show higher homozygosity (and fixation) in a smaller population than in a larger one.

To estimate *δ* and *d* we used algorithms that account for overlapping generations derived in (García-Dorado et al. 2016), and the corresponding software PURGd v2.1.5 that analyzes data for fitness traits in pedigreed individuals. This software searches the estimates of *d, δ*, and *W*_0_, as well as the effects of additional factors, using a numerical approach that minimizes the residual sum of squares of the fit between the observed fitness values and those expected in the IP model (García-Dorado 2012). An additional estimate of the expected fitness of the reference non-inbred population was obtained as the mean fitness of non-inbred individuals with non-inbred ancestors (*W’*_0_). The effect of purging of the inbreeding load ascribed to maternal effects (*δ*_*M*_) was also explored in the analyses, together with the three additional factors mentioned above: *S*, *POM*, and *YOB*. Models with all possible combinations of the above parameters were analyzed, but *POM* and *YOB* were never considered together as they are very associated with each other.

The statistical significance of purging is given by the *P* value of the *χ*^2^ test for the log-likelihood ratio of the purging model relative to a model assuming no purging (i.e., using *d* = 0 when computing *g*_*i*_) (Casella and Berger 2002). PURGd was executed with 100 independent runs per study case (i.e., 100 replicates), 10^3^ parameter evaluations per iteration and 100 iterations to convergence. For the cases where purging was non-significant, the significance of *δ* was tested using the same log-likelihood ratio procedure to compare the analysis estimating *δ* assuming *d* = 0 to another analysis assuming *d* = 0 and *δ* = 0, and the same additional factors as in the best fit model but with no maternal effects.

### Proposing a minimum number of generations to observe purging

Here we obtained an expression (*t*_*m*_) that can be helpful to approximate the minimum number of generations required before purging effects can be detected (*t*_*m*_). We used the ancestral inbreeding coefficient (*F*_*a*_) (Ballou 1997), defined as the fraction of the genome that has been exposed to inbreeding in at least one ancestor. Its expected value after *t* generations with constant effective population size *N* can be computed as $F_a = 1 - \left( {1 - \frac{1}{{2N}}} \right)^{\frac{1}{2}t\left( {t - 1} \right)}$ (López-Cortegano et al. 2018), so that it monotonically increases with *t* and asymptotically approaches 1. The first derivative of this function has a maximum value that represents the generation *t*_*m*_ at which ancestral inbreeding is expected to increase fastest, providing increased opportunities for purging to operate. We assume that detecting purging before *t*_*m*_ generations is unlikely as there has been only very limited purging opportunities. This *t*_*m*_ value can be obtained by equating the second derivative of *F*_*a*_ to zero. Thus, solving $F_{a}^{\prime\prime} = 1 + \left( {t - \frac{1}{2}} \right)^2{{{\rm{log}}}}\left( {1 - \frac{1}{{2N}}} \right) = 0$ for the number of generations *t*, we find:

$$t_m = \frac{1}{2} + \sqrt {\frac{1}{{{{{\rm{log}}}}\,\left( {1 + \frac{1}{{2N - 1}}} \right)}}}$$

which can be approximated as follows:

$$t_m \approx \sqrt {2N} ,$$

or

$$t_m \approx \sqrt {2N} + 1$$

in the absence of self-fertilization. The accumulation of detectable purging effects after generation *t*_*m*_ can still take a long time, particularly for alleles with mild or small *d* values that need to be repeatedly exposed in homozygosis to be purged.

## Results

### Population demography

Demographic results are given in Table 2. Pedigree completeness was above 95% for all four species. The estimates of the effective population size (*N*_*e*_), computed using the per generation rate of inbreeding of individuals in the TP (Gutiérrez et al. 2008), are small (*N*_*e*_ < 40). These estimates of *N*_*e*_ were virtually identical to those computed solving for *N* the classical non-overlapping generations expression for the average inbreeding in the last cohort, $F = 1 - \left( {1 - \frac{1}{{2N}}} \right)^t$ (Appendix S3). Based on these *N*_*e*_ estimates, the *t*_*m*_ expression giving the number of generations before which there is little opportunity for purging detection gives *t*_*m*_ = 3.8, 6.3, 9.9, and 5.7 for the four populations (ordered as in Table 2). Although *t*_*m*_ assumed constant population size, comparing these values with the corresponding *EqG* suggests that the period studied is too short for purging to be detected in the case of *G. dorcas*.

The number of founders of the TP was very small in all but one species. The smallest effective number of founders relative to the actual number (*N*_*ef*_/*AN*_*f*_) corresponded to *G. dorcas*, followed by *N. dama*, which implies more unbalanced contributions of the actual founders. In agreement with these observations, the ratio of *N*_*e*_ to the average number of individuals per equivalent generation (i.e., *N*_*e*_/(*N*/*EqG*)) was larger for *A. lervia* and *G. cuvieri* (0.89) than for *G. dorcas* and *N. dama* (0.67 and 0.65, respectively). Additional measures for unbalanced ancestor contributions are shown in Appendix S3 (Supplementary Table S2 and Supplementary Fig. S1). All these results suggest that population management performed with *G. cuvieri* and in *A. lervia* at La Hoya preserved their genetic diversity better than in the other two species. Results found in *G. dorcas* are unsurprising, as a considerable proportion of the adults were not used as breeders during the first years of the program (Abáigar 1993). In the case of *N. dama*, the more likely explanation is that, from 1971 to approximately 2006, breeding strategies were designed using the pedigree available by that time (Cano 1991) and considering as founders the animals arriving La Hoya in the 1970s. Ruiz-López et al. (2010) demonstrated, using molecular techniques, that some of these individuals were relatives, and they reconstructed a deeper pedigree with five unknown ancestors that was used for breeding design thereafter (Espeso 2018) as well as for the present analysis of inbreeding and purging. This illustrates how in captive breeding programs the maintenance of genetic diversity can benefit both from extending breeding opportunities to as many individuals as possible (males and females) within the managed population, and from a high-quality pedigree back to the actual population founders.

### Inbreeding and purging

Estimates of the IP parameters from the best fit model for survival *W*_*S*_ are shown in Table 3. Standard errors over different runs for analysis of the same data set were at most 0.01, indicating good convergence for the estimation numerical approach. No significant purging was detected for *A. lervia* nor *G. dorcas* while the estimate of the purging coefficient was significantly higher than 0 for *G. cuvieri* and *N. dama*. Results for the whole set of models analyzed are given in Supplementary Tables S3–S6. For the two species with significant purging (*G. cuvieri* and *N. dama*), the models where *d* was estimated from the data always fitted better than the analogous model where *g*_*i*_ was computed assuming *d* = 0, as shown by the small *P* values of the corresponding *d* estimates (Supplementary Tables S3–S6). In these two species, both the estimates of *d* and of the overall inbreeding load (*δ* + *δ*_*M*_*)* obtained in the different models analyzed were similar to those of the best fit model, particularly when the corresponding AICc values were close to those of the best fit.

**Table 3:** Inbreeding-purging parameters estimated for *W*_*S*_: purging coefficient (*d*), *P* value for the purging coefficient, rate of inbreeding depression (*δ)* ascribed to the individual’s genotype, maternal rate of inbreeding depression *(δ*_*M*_), sex effect for males (*S*), effect of the period of management with regular veterinary care (*POM*), and estimate of the trait average for the reference non-inbred population (*W*_0_).

Species	*d*	*P* value	*δ*	*δ*_*M*_	*S*	*POM*	*W*_0_	*W’*_0_
*A. lervia*	0.08	0.34	0.26	NA	−0.06	0.13	0.90	0.75 (0.22)
*G. cuvieri*	0.48	2.13e–3	0.67	0.66	−0.10	NA	0.96	0.90 (0.06)
*G. dorcas*	0.48	0.31	0.51	0.25	−0.06	0.11	0.81	0.78 (0.02)
*N. dama*	0.23	2.78e–2	0.88	0.36	−0.05	NA	0.94	0.87 (0.09)

NA values indicate that the factor was not selected in the best fit model. The last column gives the trait average for non-inbred individuals with non-inbred ancestors and its empirical standard error [W'_0_ (SE)].

The estimates of the non-maternal inbreeding load (*δ*) were generally small, but adding up both the direct and maternal components gives an overall inbreeding load larger than one for *G. cuvieri* and *N. dama* (*δ* + *δ*_*M*_ = 1.33 and 1.24, respectively). Classically, inbreeding load has often been estimated by comparing the average of a fitness trait in a non-inbred and an inbred group. In that context, the inbreeding coefficient of mothers and offspring in the inbred group is usually relatively similar, so that a pooled estimate of the inbreeding load can be obtained including both the direct and the maternal components. Pedigree data, however, allow analysis of the association between *F* and *W* at the individual level, where *F* can be very different in an individual and its mother. Our results show that estimating the survival inbreeding load from pedigree data requires accounting for maternal effects to avoid missing the inbreeding load ascribed to deleterious alleles that, when expressed in homozygosis in the mother, reduce the survival of the offspring. The individual sex effect (*S*) was selected as a relevant parameter in the best fit model for all four species, survival being always smaller in males (S ≤ –0.05). The introduction of regular veterinary care (*POM*) had a positive effect on survival when it was present in the best model (Table 3). The year of birth (*YOB*) was never included in the best model.

Regarding the two species where purging was found not to be significantly different from zero, an analysis performed assuming no purging (i.e., setting *d* = 0 to compute *g*_*t*_) gave higher likelihood when *δ* was estimated than when *δ* was set to zero, but only the *δ* estimate of *G. dorcas* was significantly higher than zero (*P* value = 0.02). The estimate for *A. lervia* was not (*P* value = 0.25), implying no evidence of inbreeding depression.

**Fig. 1:**
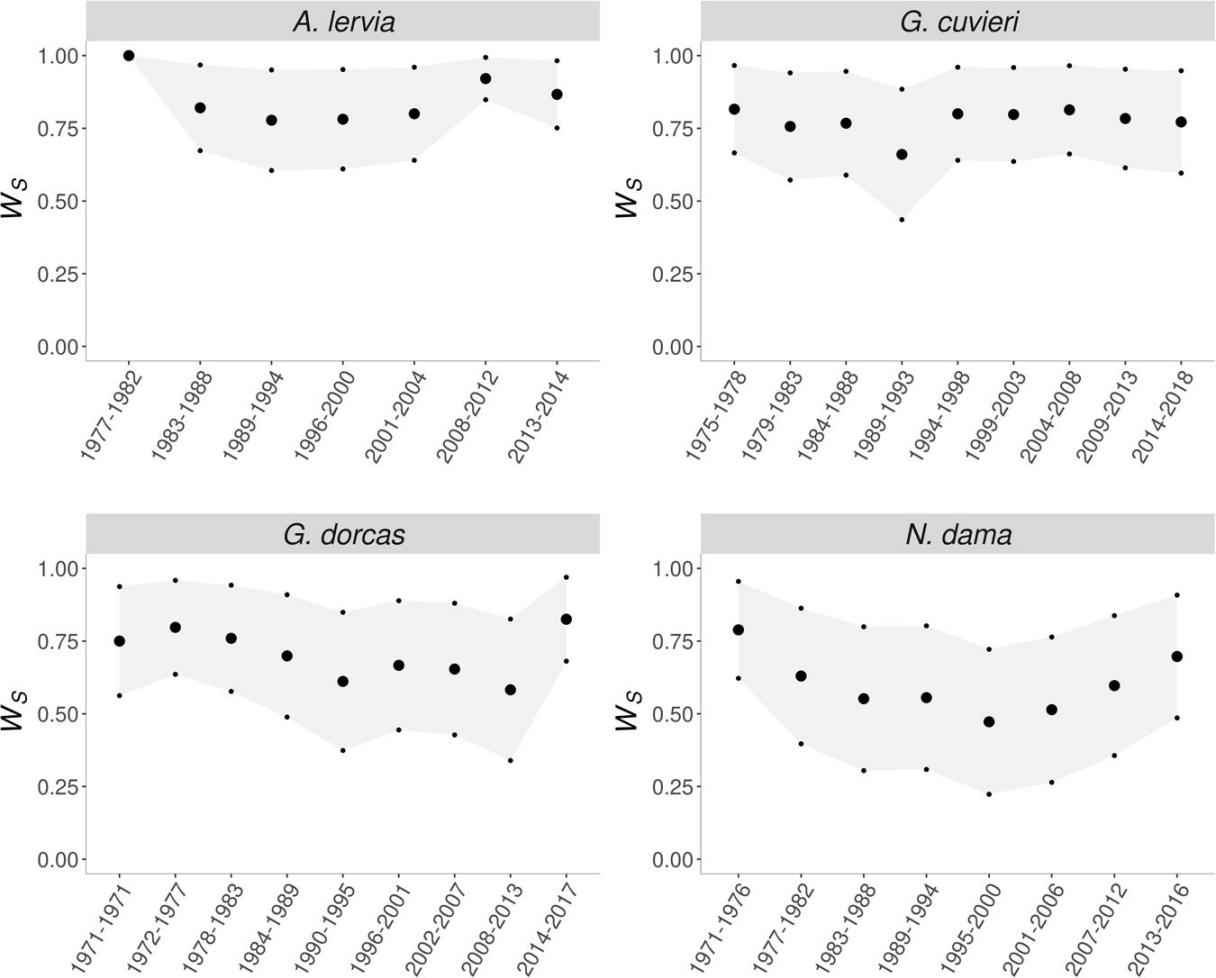
Evolution of early survival (*W*_*S*_). Large dots represent mean *W*_*S*_, while small dots correspond to the mean value plus or minus one standard error. The amplitude of the intervals (cohorts) is calculated as the number of years recorded since the first survival data were available over the maximum number of equivalent complete generations rounded to the upper integer and years with non-available records are excluded.

**Fig. 2:**
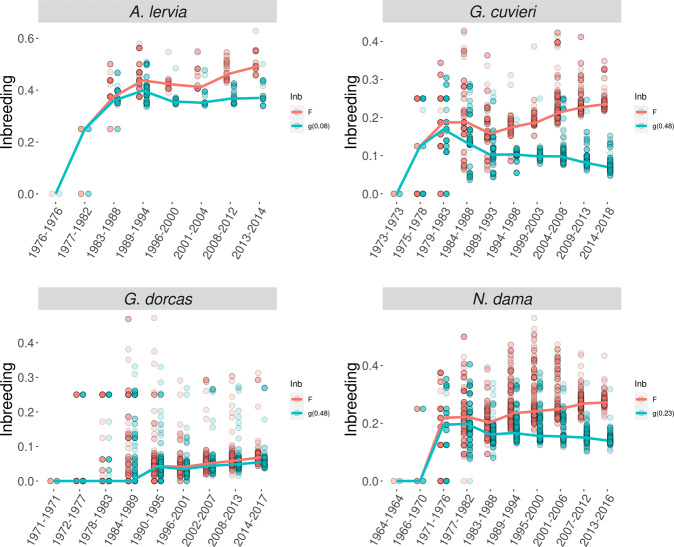
Evolution of the standard (*F*, red) and purged (*g*, green) inbreeding coefficients through time. Darker dots indicate larger number of observations. The lines join the median survivals of consecutive intervals. The amplitude of the intervals (cohorts) is calculated as the number of years recorded divided by the maximum number of equivalent complete generations estimated rounded to the upper integer.

The evolution of average survival through generations did not show a clear pattern of inbreeding depression (Fig. 1), and only *N. dama* seemed to show a consistent initial reduction followed by a partial recovery in survival, as expected from purging. However, this is not surprising, since average inbreeding did not increase with generations as expected under a non-managed situation with constant effective size. In fact, inbreeding first increased abruptly due to the small number of founders but then declined due to mating management. In addition, it later increased at a rate that was slowed due to increased population size (Fig. 2). Furthermore, there is one generation gap in the consequences of this process on maternal and direct components of depression. Finally, in the case of *A. lervia* and *G. dorcas* there was also an environmental effect due to the establishment of regular veterinary care. These factors can mask the effect of purging in Fig. 1, although, since they were included in the analyzed models, should not affect the IP estimates.

Results of the IP analysis for female productivity *W*_*P*_ can be found in Supplementary Table S7. For this trait, the purging coefficient was never significant. The inbreeding load was significantly higher than zero for all species (*P* value < 10^−8^) and the overall value, computed adding both the direct and maternal components, was large, ranging from 4.6 to 6.7.

The evolution of individual inbreeding (*F*) and purged inbreeding [*g*, computed using the corresponding algorithms (García-Dorado et al. 2016) and the estimates of *d* reported in Table 3], are given in Fig. 2. The values for *g* became smaller than those for *F* after a few generations, again with the exception of *G. dorcas*, the largest population. In any case, *g* should be assumed to be non-significantly different from *F* for *A. lervia* and for *G. dorcas*, as *d* was non-significantly different from 0.

Regarding the TP, the average inbreeding was similar in *G. cuvieri* and in *N. dama* (*F* = 0.24 and *F* = 0.28, respectively) but the average purged inbreeding was considerably smaller in *G. cuvieri* due to the higher purging coefficient (*g*_(*d* = 0.48)_ = 0.08 in G. *cuvieri* and *g*_(*d* = 0.23)_ = 0.15 in *N*. *dama*). Thus, by the end of the period recorded in the pedigrees of these two species, the mean *g* value had been reduced to between 30 and 50% the mean of *F*, implying that purging had caused a similar reduction in the frequencies of the deleterious alleles responsible for the initial inbreeding load.

## Discussion

We have analyzed the inbreeding-purging process in four captive populations of different ungulate species with effective sizes ranging 4–40 and with available pedigrees as well as survival and productivity records. This allows us to explore the role of inbreeding and purging in determining the evolution of fitness traits in a range of scenarios relevant in the context of conservation.

In *A. lervia* (*N*_*e*_ ≈ 4), purging is expected only for the most severely deleterious alleles (those giving *dN*_*e*_ > 1, which implies *d* > 0.25 as, for example, in completely recessive alleles with deleterious homozygous disadvantage *s* > 0.5). Thus, it could be that purging has not been detected for this species because such severely deleterious alleles had been purged during the demographic decline in the wild, before the foundation of the captive population. This would be consistent with the low and non-significant inbreeding load estimated in this species. It is also possible that these estimates are non-significant due to the relatively small number of individuals available.

*G. cuvieri* and *N. dama* have significant initial inbreeding loads that, adding up the direct and maternal components, is about 1.25 in both cases, which is on the order of other estimates published for captive populations (Ralls et al. 1988). Since in both species *N*_*e*_ > 10, purging should be efficient against less severely deleterious alleles than in *A. lervia* (*d* > 0.1). Purging is detected for both species with very low *P* values. This result is in agreement with Moreno et al. (2015), who suggested that purging had occurred in *G. cuvieri* as they found an increased juvenile survival parallel to an increased inbreeding coefficient. The relative contribution of severe and mild deleterious effects to the inbreeding load of populations is under a scientific debate with direct implications in conservation biology (Ralls et al. 2020, Kyriazis et al. 2021, Pérez-Pereira et al. 2021). The large *d* estimates obtained in our analysis indicate that a substantial fraction of the initial inbreeding load is being purged under modest effective population sizes, implying that such substantial fraction is due to relatively severe deleterious mutations in these two populations. As far as we are aware, these are the first estimates of this purging parameter obtained in managed, non-experimental populations. Previous estimates of *d* were obtained in *D. melanogaster* bottlenecked populations, first for egg-to-pupae viability in lines with *N*_*e*_ = 6 or 12 under noncompetitive conditions (*d* = 0.09, Bersabé and García-Dorado 2013), and second in lines with higher *N*_*e*_ ≈ 40–50 under more competitive conditions, giving a larger estimate of *d*, of the order of that estimated in these two ungulate endangered species (*d* ≈ 0.3, López-Cortegano et al. 2016).

Regarding *G. dorcas*, given its larger population size, purging is expected even against alleles with mild recessive component of the deleterious effect (*d* > 0.025). However, although a significant (if modest) inbreeding load was estimated, no significant purging was detected. Nevertheless, the number of equivalent complete generations by the end of the pedigree (*EqG* = 7) was smaller than our proposed minimum number of generations required to detect purging (*t*_*m*_ = 10). This suggests that, due to the large size of this population, more generations are needed to detect purging.

The results above support the use of *t*_*m*_ to get an approximate idea about when a pedigree is too shallow for purging to be detected. Should the number of generations available be larger than *t*_*m*_, IP predictions could additionally be computed to search the *d* values that can be expected to produce detectable purging. Supplementary Fig. S3 shows that the true number of generations required to detect purging becomes increasingly larger than *t*_*m*_ for alleles with smaller *d* values, as they suffer weaker purging each time they are exposed in homozygosis. The *t*_*m*_ approach helps to understand the failure of many studies to detect purging. Such is the case of the extensive meta-analyses on 119 zoo populations by Boakes et al. (2007), where the median *N*_*e*_ value was 22.6 while the median number of generations was *t* = 3 meaning that, for most species, at least 5 more generations were needed before purging could be detectable. On the contrary, and in agreement with this *t*_*m*_ approach, purging was experimentally detected in lines of *D. melanogaster* with *N*_*e*_ = 43 (i.e., *t*_*m*_ ≈ 10) where, after an initial period of inbreeding depression, fitness experienced a substantial recovery beginning between generations 10 and 20 (López-Cortegano et al. 2016).

A reason why detecting purging in captive populations is challenging is that a fitness rebound can also be due to adaptation to captive conditions or to environmental effects, such as those derived from improved husbandry (Clifford et al. 2007). In fact, this might have been the case in Speke’s gazelle breeding program, where the observed rebound of fitness was first ascribed to purging (Templeton and Read 1984, 1998), while Kalinowski et al. (2000) suggested that husbandry improvements could also be responsible for these findings. Our estimates of *d* and *δ*, however, are based on the association between the fitness trait and purged inbreeding at the individual level (*W*_*i*_*, g*_*i*_) which, in our data, is mainly expressed within cohorts while average survival showed little variation through time. In addition, the analyses included temporal factors (*YOB* or *POM*) that should have removed confounding effects from adaptation to captivity or improved husbandry. Therefore, adaptive processes or time-dependent environmental factors are not expected to have biased our IP estimates.

For productivity, the estimates of inbreeding load were high (overall inbreeding load ~5, *P* value < 10^−8^), but no significant purging was detected in any species (Supplementary Table S7). Note, however, this trait was assayed only in females, and only in those that had completed their reproductive life by the end of the pedigree (see Methods). This implies less statistical power than for survival and, more importantly, fewer generations for purging to occur (only about six generations in the case of *G. cuvieri* and *N. dama*, for which *t*_*m*_ ≈ 6). Thus, detecting purging for productivity was in fact hardly expected.

Genomic data are useful to measure inbreeding and, therefore, to estimate inbreeding depression (Kardos et al. 2018). The footprint of slow purging has been detected as a reduction of the genomic burden of putatively deleterious alleles in populations that suffered historical bottlenecks (Xue et al. 2015; Grossen et al. 2020). In line with these advances, inferred functional genetic variation observed at the genomic level has been recently proposed as a tool to select individuals in conservation biology (Kyriazis et al. 2021; Teixeira and Huber 2021, but see Ralls et al. 2020; García-Dorado and Caballero 2021). However, the magnitude of the deleterious effect or of the potentially adaptive effects of most genomic variants cannot at present be inferred with any certainty, and there is yet no way to infer from genomic information the amount of purging accumulated in the ancestors of an individual. Therefore, the analysis of pedigreed fitness data is an essential tool to evaluate the fitness impact of purging during ex situ breeding and its conservation impact, although additional genomic analysis can be helpful as, for example, to infer previous demographic and selective processes.

The evaluation of the efficiency of purging may help to determine the minimum viable population size (MVP) that has been proposed as a rule of thumb in conservation guidelines. Classically, genetic considerations lead to a 50/500 MVP rule being proposed for the effective population size, where the lower figure was aimed to prevent excessive inbreeding depression in the short to medium term, while the larger one was intended for the long-term preservation of adaptive potential (Franklin 1980; Jamieson and Allendorf 2012). There has been some debate on the appropriateness of updating the rule to 100/1000 which, regarding the first figure, was based on the high estimates of the inbreeding load reported in the wild (*δ* ≈ 6, O’Grady et al. 2006; Frankham et al. 2014a, b; Franklin et al. 2014). However, our results illustrate that purging can be relevant even in populations with effective size scarcely over 10, where no evidence of purging during the first generations after a bottleneck is not indicative that purging will not be able to induce substantial fitness recovery later on. Thus, although increasing the size of endangered populations should always be a major aim, considering purging can lead to a more flexible value for the lower MVP figure regarding inbreeding depression. Although attention needs to be paid to the threat derived from the loss of genetic diversity and adaptive potential, these results encourage conservation efforts even on populations that seem stalled in a too small census (García-Dorado 2015; Caballero et al. 2017).

Due to the nature of the data, our results are particularly relevant to ex situ conservation. Since the facility at La Hoya allowed most surviving females to breed every year, our populations were maintained with the main priority of increasing population size. This strategy contrasts with the Minimum Kinship protocol (MK), where breeding adults are chosen to minimize the average coancestry of the progeny in order to maximize genetic diversity (Ballou and Lacy 1995; Fernández and Toro 1999). MK is recommended to preserve adaptive potential and to slow adaptation to captive conditions, but implies setting the number of breeding offspring contributed by each parent, which becomes independent of the parents’ reproductive fitness. This leads to some relaxation of natural selection with different consequences for survival and reproductive fitness: MK is favorable regarding survival, since both inbreeding and purging (which occurs just within families) are slowed; MK relaxes purging for reproductive fitness, so that it could be risky in the medium to long term (García-Dorado 2012). On the contrary, the managing protocol followed at La Hoya should have allowed genetic purging for both survival and reproductive fitness. The detection of purging when the data were appropriate (survival in Cuvier´s and Dama gazelles), together with recent evidence that productivity has not declined in Cuvier’s gazelle despite its high initial inbreeding load (Moreno et al. 2020), suggests that purging may have also occurred for productivity but was not detected due to the limited sample size and pedigree depth.

Our results illustrate that, when it is necessary to decide which individuals are going to breed, some compromise may be necessary between two extreme options: (i) allowing breeders to contribute breeding offspring proportionally to their observed reproductive fitness in order to favor purging; (ii) choosing breeders according to MK to maximize genetic diversity and slow inbreeding. Such compromise should be dependent upon the population size, the inbreeding load (particularly that for reproductive traits), the average reproductive potential, and the time horizon of the captive breeding program. If the population size is so small that inbreeding depression and loss of adaptive potential are an immediate concern, MK is to be advised. If the effective population size is on the order of several tens, it could be advisable to assign some weight to productivity when choosing breeding individuals instead of relying exclusively on minimizing average kinship or even, in some cases, to move toward a breeding system with no genetic management, as under reintroduction. This could prevent the medium to long-term consequences of the relaxation of purging for reproductive traits.

Selection of breeding individuals and the management of their mating have different consequences and should better be established through a two-step protocol (Fernández and Caballero 2001). Mating management is usually intended to avoid inbred mating, which is expected to reduce homozygosis. This implies less inbreeding depression in the first generations, but also less-efficient purging and no additional long-term protection of adaptive potential, as well as some increase in genetic drift (Caballero and Toro 2000). This mating management should in principle be applied as far as population growth is compromised by immediate fitness inbreeding depression, as in a very small population with high inbreeding load. In the case of our populations, from the mid-1980s onwards, matings at La Hoya have been managed to minimize coancestry between pairing individuals in order to reduce inbreeding in the offspring, a strategy that should reduce the efficiency of purging. However, based on the trend of more-inbred mothers to produce more surviving daughters than sons (Moreno et al. 2011) and in agreement with other authors (Tella 2001), beginning in 2006 more-inbred females were promoted to be the ones mating males with lower coancestry in *G. cuvieri* (Moreno et al. 2015). This implies that: (i) they contribute offspring with lower inbreeding and, therefore, higher survival; (ii) due to their mothers’ high inbreeding, these offspring are more purged and, those surviving, also show a higher female/male ratio. Thus, very likely, this method, besides producing a more convenient sex ratio for the management of polygynous species, has improved purging opportunities. The high purging detected in this population suggests that the reduction of purging from minimum coancestry mating has been mitigated by favoring the breeding contribution of more-inbred females, which should have also contributed to the maintenance of genetic diversity (Caballero and Toro 2000). In ex situ conservation programs, alternative mating strategies have been proposed to improve purging while controlling inbreeding depression, as in the case of circular mating (Theodoru and Couvet 2015). However, when considering such strategies, the extinction risk from short-term inbreeding depression should be carefully evaluated considering the population reproductive potential (Caballero et al. 2017).

As Tudge (1991) asserts, the proper end point of captive breeding is reintroduction. So, the success of the above recommendations in captive breeding programs depends on how far purging occurring in captive conditions will reduce the inbreeding depression expressed in the wild. Obviously, purging during ex situ conservation is not expected to act upon traits that are not expressed in captivity but that could be fitness components in the wild. For example, the inbreeding load for heat shock resistance in *Drosophila* was not purged in lines that had been maintained in stable lab conditions (Bundgaard et al. 2021). However, mutation is expected to produce unconditionally deleterious alleles much more often than alleles that are deleterious in some conditions but advantageous in others. This is not in contradiction with the notion that adaptation to captive conditions should entail some maladaptation in the wild, but implies that the genetic basis of adaptive tradeoffs may contribute a small fraction of the inbreeding load. Thus, the purging of such unconditional deleterious alleles occurred ex situ should, to some extent, be expressed in the wild. Furthermore, it is usually considered that the deleterious effects of mutations tend to be larger when expressed in harsh environments (Halligan and Keightley 2009), accounting for a larger inbreeding load when measured in wild conditions (Keller and Waller 2002; Armbruster and Reed 2005; Fox and Reed 2011). For example, López-Cortegano et al. (2016) found that purging under competitive conditions was efficient against inbreeding depression expressed for both competitive and noncompetitive fitness, and their results suggested that the larger inbreeding load estimated in competitive conditions could be mainly ascribed to the same deleterious alleles as in noncompetitive ones but with larger effects. In such situation, although purging is expected to be more efficient if it occurs in the wild, even purging in captivity could remove a substantial fraction of the inbreeding load to be expressed in wild conditions (Swindell and Bouzat 2006b). In our case, the successful recent reintroduction of *G. cuvieri* in Tunisia (Moreno et al. 2020) has produced a healthy population with vigorous productivity since 2016 and that shows no apparent sign of inbreeding depression, suggesting that purging in captivity has reduced the inbreeding depression expressed in wild conditions Nevertheless, additional research is required regarding the consequences of purging in captive conditions on population survival after reintroduction.

Overall, the large *d* estimates obtained for survival in *G. cuvieri* and *N. dama*, the two species where *N*_*e*_ and the time scale provided opportunity for purging detection, illustrate that a large fraction of the inbreeding load can be purged when breeding contributions are governed by natural selection. Our results suggest that, during ex situ conservation, it may be appropriate to progressively move to a breeding strategy allowing for purging on productivity based either on management protocols that take this trait into account or on reduced breeding intervention allowing selection to act. The latter option encourages early reintroduction efforts, with the additional advantage of allowing purging to occur under wild conditions. However, this should be considered only after an initial ex situ phase of demographic recovery (say, *N*_*e*_ above a few tens), so that the loss of adaptive potential and the early inbreeding depression are not threatening and purging is reasonably efficient.

## Supplementary information

Supplementary Material

## Data Availability

Studbooks are publicly available at http://www.eeza.csic.es/es/programadecria.aspx. PURGd is available for download at https://gitlab.com/elcortegano/PURGd, where the processed pedigrees as well as additional C++ code and R scripts used to estimate parameters such as the effective population size can be found. PURGd is also available at https://www.ucm.es/gfm/mecanismos-geneticos.

## References

[CR1] Abáigar T (1993). Gazella dorcas neglecta Studbook.

[CR2] Armbruster P, Reed DH (2005). Inbreeding depression in benign and stressful environments. Heredity.

[CR3] Ávila V, Amador C, García-Dorado A (2010). The purge of genetic load through restricted panmixia in a Drosophila experiment. J Evol Biol.

[CR4] Ballou JD (1997). Ancestral inbreeding only minimally affects inbreeding depression in mammalian populations. J Hered.

[CR5] Ballou JD, Lacy RC, Ballou JD, Gilpin M, Foose TJ (1995). Identifying genetically important individuals for management of genetic variation in pedigreed populations. Population management for survival and recovery: analytical methods and strategies in small population conservation.

[CR6] Bersabé D, García-Dorado A (2013). On the genetic parameter determining the efficiency of purging: an estimate for Drosophila egg-to-pupae viability. J Evol Biol.

[CR7] Beudels RC, Devillers P, Lafontaine RM, Devillers-Terschuren J, Beudels MO (2005). Sahelo-Saharan antelopes. Status and perspectives. CMS Technical Series Publication, N° 11.

[CR8] Boakes EH, Wang J, Amos W (2007). An investigation of inbreeding depression and purging in captive pedigreed populations. Heredity.

[CR9] Boichard D, Maignel L, Verrier E (1997). The value of using probabilities of gene origin to measure genetic variability in a population. Genet Sel Evol.

[CR10] Bouzat JL (2010). Conservation genetics of population bottlenecks: the role of chance, selection, and history. Conserv Genet.

[CR11] Bundgaard J, Loeschcke V, Schou MF, Bijlsma KR (2021) Detecting purging of inbreeding depression by a slow rate ofinbreeding for various traits: the impact of environmental and experimental conditions. Heredity 127(1):10–20. 10.1038/s41437-021-00436-7 10.1038/s41437-021-00436-7 PMC8249611 33903740

[CR12] Byers DL, Waller DM (1999). Do plant populations purge their genetic load? Effects of population size and mating history on inbreeding depression. Ann Rev Syst Ecol.

[CR13] Caballero A, Toro M (2000). Interrelations between effective population size and other pedigree tools for the management of conserved populations. Genet Res.

[CR14] Caballero A, Bravo I, Wang J (2017). Inbreeding load and purging: implications for the short-term survival and the conservation management of small populations. Heredity.

[CR15] Cano M (1991). El antílope Mohor, Gazella (Nanger) dama mhorr Bennett 1832 en cautividad.

[CR16] Cano M (1988) Sobre las poblaciones de ungulados del Parque de Rescate de la Fauna Sahariana durante el período 1971-1986. Boletín del Instituto de Estudios Almerienses. Volumen Extraordinario: Homenaje a Manuel Cano Gea, p 281–292

[CR17] de Cara M, Villanueva B, Toro MA, Fernández J (2013). Purging deleterious mutations in conservation programmes: combining optimal contributions with inbred matings. Heredity.

[CR18] Casella G, Berger RL (2002) Statistical inference, 2nd ed. Duxbury Press, Belmont

[CR19] Cassinello J (1998). El arrui sahariano. Un caprino ancestral en Almería.

[CR20] Cassinello J (2005). Inbreeding depression on reproductive performance and survival in captive gazelles of great conservation value. Biol Conserv.

[CR21] Cassinello J, Alados CL (1996). Female reproductive success in captive *Ammotragus lervia* (Bovidae, Artiodactyla). Study of its components and effects of hierarchy and inbreeding. J Zool Lond.

[CR22] Cassinello J, Gomendio M, Roldan ERS (2001). Relationship between coefficient of inbreeding and parasite burden in endangered gazelles. Conserv Biol.

[CR23] Charlesworth B (2018). Mutational load, inbreeding depression and heterosis in subdivided populations. Mol Ecol.

[CR24] Charlesworth B, Willis JH (2009). The genetics of inbreeding depression. Nat Rev Genet.

[CR25] Clifford DL, Woodroffe R, Garcelon DK, Timm SF, Mazet JAK (2007). Using pregnancy rates and perinatal mortality to evaluate the success of recovery strategies for endangered island foxes. Anim Conserv.

[CR26] Crnokrak P, Barrett SCH (2002). Purging the genetic load: a review of the experimental evidence. Evolution.

[CR27] Crow JF, Kojima KI (1970). Genetic loads and the cost of natural selection. Mathematical topics in population genetics.

[CR28] Crow JF (2008). Mid-century controversies in population genetics. Annu Rev Genet.

[CR29] Espeso G (2018) International Mhorr Gazelle Studbook. Nanger dama mhorr. http://www.eeza.csic.es/documentos/STBDA_18.txt

[CR30] Fernández J, Toro MA (1999). The use of mathematical programming to control inbreeding in selection schemes. J Anim Breed Genet.

[CR31] Fernández J, Caballero A (2001). A comparison of management strategies for conservation with regard to population fitness. Conserv Genet.

[CR32] Fox CW, Reed DH (2011). Inbreeding depression increases with environmental stress: an experimental study and meta‐analysis. Evolution.

[CR33] Frankham R (2010). Inbreeding in the wild really does matter. Heredity.

[CR34] Frankham R, Bradshaw CJA, Brook BW (2014). Genetics in conservation management: Revised recommendations for the 50/500 rules, Red List criteria and population viability analyses. Biol Conserv.

[CR35] Frankham R, Bradshaw CJA, Brook BW (2014). 50/500 rules need upward revision to 100/1000 – Response to Franklin et al. Biol Conserv.

[CR36] Franklin LR, Allendorf FW, Jamieson LG (2014). The 50/500 rule is still valid – Reply to Frankham et al. Biol Conserv.

[CR37] Franklin LR (1980) Evolutionary change in small populations. In: Soulé ME, Wilcox BA (eds) Conservation biology: An evolutionary-ecological perspective. Sinauer Associates, Sunderland, p. 135–150

[CR38] García-Dorado A (2012). Understanding and predicting the fitness decline of shrunk populations: inbreeding, purging, mutation, and standard selection. Genetics.

[CR39] García-Dorado A (2015). On the consequences of ignoring purging on genetic recommendations for minimum viable population rules. Heredity.

[CR40] García-Dorado A, Wang J, López-Cortegano E (2016). Predictive model and software for inbreeding-purging analysis of pedigreed populations. G3.

[CR41] García-Dorado A, Caballero A (2021) Genetic diversity as a useful guide for conservation genetics. Conserv Genet. 10.1007/s10592-021-01384-9

[CR42] Grossen C, Guillaume F, Keller LF, Croll D (2020). Purging of highly deleterious mutations through severe bottlenecks in Alpine ibex. Nat Commun.

[CR43] Gutiérrez JP, Cervantes I, Goyache F (2009). Improving the estimation of realized effective population sizes in farm animals. J Anim Breed Genet.

[CR44] Gutiérrez JP, Cervantes I, Molina A, Valera M, Goyache F (2008). Individual increase in inbreeding allows estimating effective sizes from pedigrees. Genet Sel Evol.

[CR45] Halligan DL, Keightley PD (2009). Spontaneous mutation accumulation studies in evolutionary genetics. Annu Rev Ecol Evolution Syst.

[CR46] Hedrick PW (1994). Purging inbreeding depression and the probability of extinction: full-sib mating. Heredity.

[CR47] Hedrick PW, Kalinowski ST (2000). Inbreeding depression in conservation biology. Annu Rev Ecol Syst.

[CR48] Hedrick PW, García-Dorado A (2016). Understanding inbreeding depression, purging, and genetic rescue. Trends Ecol Evol.

[CR49] Ibáñez B, Moreno E, Barbosa A (2011). No inbreeding effects on body size in two captive endangered gazelles. Mamm Biol.

[CR50] Ibáñez B, Cervantes I, Gutiérrez JP, Goyache F, Moreno E (2014). Estimates of direct and indirect effects for early juvenile survival in captive populations maintained for conservation purposes: the case of Cuvier’s gazelle. Ecol Evol.

[CR51] IUCN (2019) The IUCN Red List of Threatened Species. Version 2019-3. http://www.iucnredlist.org

[CR52] Jamieson IG, Allendorf FW (2012). How does the 50/500 rule apply to MVPs?. Trends Ecol Evol.

[CR53] Kalinowski ST, Hedrick PW, Miller PS (2000). Inbreeding depression in the Speke’s gazelle captive breeding program. Conserv Biol.

[CR54] Kardos M, Åkesson M, Fountain T, Flagstad Ø, Liberg O, Olason P (2018). Genomic consequences of intensive inbreeding in an isolated wolf population. Nat Ecol Evolution.

[CR55] Keller LF, Waller DM (2002). Inbreeding effects in wild populations. Trends Ecol Evol.

[CR56] Kennedy ES, Grueber CE, Duncan RP, Jamieson IG (2014). Severe inbreeding depression and no evidence of purging in an extremely inbred wild species – The Chatham island black robin. Evolution.

[CR57] Kyriazis CC, Wayne RK, Lohmueller KE (2021). Strongly deleterious mutations are a primary determinant of extinction risk due to inbreeding depression. Evol Lett.

[CR58] Lande R (1994). Risk of population extinction from fixation of new deleterious mutations. Evolution.

[CR59] Latter B, Mulley J, Reid D, Pascoe L (1995). Reduced genetic load revealed by slow inbreeding in *Drosophila melanogaster*. Genetics.

[CR60] Leberg PL, Firmin BD (2008). Role of inbreeding depression and purging in captive breeding and restoration programmes. Mol Ecol.

[CR61] López-Cortegano E, Vilas A, Caballero A, García-Dorado A (2016). Estimation of genetic purging under competitive conditions. Evolution.

[CR62] López-Cortegano E, Bersabé D, Wang J, García-Dorado A (2018). Detection of genetic purging and predictive value of purging parameters estimated in pedigreed populations. Heredity.

[CR63] Moreno E, Ibáñez MB, Barbosa A (2011). Mother traits and offspring sex in two threatened gazelles species incaptivity. J Nat Conser.

[CR64] Moreno E, Pérez-González J, Carranza J, Moya-Laraño J (2015). Better fitness in captive Cuvier´s gazelle despite inbreeding increase: Evidence of purging?. PLoS One.

[CR65] Moreno E, Jebali A, Espeso G, Benzal J (2020) Reintroducing Cuvier’s gazelle. Better than expected from captive-bred founders. Glob Ecol Conserv 23:e01094

[CR66] Morton NE, Crow JF, Muller HJ (1956). An estimate of the mutational damage in man from data on consanguineous marriages. Proc Natl Acad Sci USA.

[CR67] O’Grady J, Brook JBW, Reed DH, Ballou JD, Tonkyn DW, Frankham R (2006). Realistic levels of inbreeding depression strongly affect extinction risk in wild populations. Biol Conserv.

[CR68] Pekkala NK, Knott E, Kotiaho JS, Puurtinen M (2012). Inbreeding rate modifies the dynamics of genetic load in small populations. Ecol Evol.

[CR69] Pérez-Pereira N, Caballero A, García-Dorado (2021) Reviewing the consequences of genetic purging on the success of rescue programs. Conserv Genet (In press)

[CR70] Ralls K, Ballou JD, Templeton A (1988). Estimates of lethal equivalents and the cost of inbreeding in mammals. Conserv Biol.

[CR71] Ralls K, Sunnucks P, Lacy RC, Frankham R (2020). Genetic rescue: A critique of the evidence supports maximizing genetic diversity rather than minimizing the introduction of putatively harmful genetic variation. Biol Conserv.

[CR72] Ruiz-López MJ, Espeso G, Evenson DP, Roldan ER, Gomendio M (2010). Paternal levels of DNA damage in spermatozoa and maternal parity influence offspring mortality in an endangered ungulate. Proc R Soc B Biol Sci.

[CR73] Swindell W, Bouzat J (2006). Reduced inbreeding depression due to historical inbreeding in *Drosophila melanogaster*: Evidence for purging. J Evol Biol.

[CR74] Swindell WR, Bouzat JL (2006). Selection and inbreeding depression: effects of inbreeding rate and inbreeding environment. Evolution.

[CR75] Tahmoorespur M, Sheikhloo M (2011). Pedigree analysis of the closed nucleus of Iranian Baluchi sheep. Small Rumin Res.

[CR76] Teixeira JC, Huber CD (2021). The inflated significance of neutral genetic diversity in conservation genetics. Proc Natl Acad Sci USA.

[CR77] Tella JL (2001). Sex-ratio theory in conservation biology. Trends Ecol Evol.

[CR78] Templeton AR, Read B (1984). inbreeding depression in a captive herd of speke’s gazelle (*Gazella spekei*). ZooBiology.

[CR79] Templeton AR, Read B (1998). Elimination of inbreeding depression from a captive population of Speke’s gazelle: validity of the original statistical analysis and confirmation by permutation testing. Zoo Biol.

[CR80] Theodoru K, Couvet D (2015). The efficiency of close inbreeding to reduce genetic adaptation to captivity. Heredity.

[CR81] Tudge C (1991). Last animals at the zoo.

[CR82] Wang J, Hill WG (1999). Effect of selection against deleterious mutations on the decline in heterozygosity at neutral loci in closely inbreeding populations. Genetics.

[CR83] Xue Y, Prado-Martinez J, Sudmant PH, Narasimhan V, Ayub Q, Szpak M (2015). Mountain gorilla genomes reveal the impact of long-term population decline and inbreeding. Science.

