# Supplementary material for: Genetic purging in captive endangered ungulates with extremely low effective population sizes
Source: Heredity (Edinb). 2021 Sep 28;127(5):433–42. doi: 10.1038/s41437-021-00473-2 (PMC8551332; doi:10.1038/s41437-021-00473-2)
Supplement: Supplementary file 1 — Supplementary Material [file 41437_2021_473_MOESM1_ESM.pdf]

SUPPLEMENTARY MATERIAL

GENETIC PURGING IN CAPTIVE ENDANGERED UNGULATES WITH EXTREMELY LOW EFFECTIVE POPULATION SIZES

Appendix S1. Species description

Appendix S2. Individuals filtered

- **Table S1.** Number of individuals during pedigree processing

Appendix S3. Complementary demographic parameters

- **Table S2.** Effective number of ancestors and number of equivalent founder genomes
- **Figure S1.** Partition of the number of founders (in %)

Appendix S4. Quantitative genetic analysis for survival

- **Figure S2.** Posterior distribution of the additive variance on 15-days survival

Tables S3-S6. PURGd results for the different models

Tables S7. Inbreeding-Purging parameters for productivity

Figure S3. Minimum number of generations for purging detection together with the evolution of the relative effect of purging on average fitness

Appendix S1. Species description

Barbary sheep (*Ammotragus lervia*) is the only caprine species housed at La Hoya. Females are fertile at about 9-10 months and males at 13-14 months. The gestation period is about 5.5 months. Twins are frequent in this species (~ 22 %; Cassinello 1998). Males are double in size than females (mean body weight in males: 82.07 kg; mean body weight in females 41.34 kg; Cassinello 1998), both sexes having horns which also differs greatly in size in favor of males. Barbary sheep use to live in different types of groups (nursery, all males, all females), but also solitary individuals are common (Gray and Simpson 1982). It is a territorial species with dispersal movements particularly accentuated in summer (Dickinson and Simpson 1980). Founder population of this species arrived La Hoya in 1975 from Western Sahara (Cano 1988). They were one male and two females.

Cuvier's gazelle (*Gazella cuvieri*) is medium-sized and sexually dimorphic, with adult males 24% heavier than adult females (average body mass of adult females: 26 kg; adult males: 34 kg; Moreno and Espeso 2008). Both sexes have horns, being lighter in females. Females are fertile at about 8–9 months and males at 12–13 months. Female reproductive senescence is around 11 years. The gestation period is about 5.5 months. Twins represent up to 39% of births (Moreno and Espeso, 2008). It lives in small, familiar groups, which typically contain fewer than eight animals (Kingdon 1997). Harems are quite frequent, with one adult male and a few adult females, accompanied by their recent youngsters (Kingdon 1997). First founders of Cuvier's gazelle captive population arrived La Hoya in 1975 from Western Sahara (Cano 1988). They were two males and two females. In 1987 a new female imported from Morocco was brought into the breeding programme (Abáigar and Cano 2005; Moreno and Espeso 2008).

Dorcas gazelle (*G. dorcas*) is one of the smallest African gazelles. Males and females are similar in size (mean body weight 15.9 kg for males and 15.7 for females; López and Abáigar 2013). Both sexes have horns, being bigger in males. Females are fertile at the age of 11-12 months and males at eight months (López and Abáigar 2013). The gestation period is 24-25 weeks (Alados 1982). Female reproductive senescence is around 11 years. There is only one calf per birth (Abáigar 2002). When conditions are harsh, dorcas gazelles live in pairs, but when conditions are more favourable, they occur in family herds with one adult male, several females and young individuals. During the breeding season, adult males tend to be territorial (Beudels et al. 2005). The founder population arrived La Hoya between 1970 and 1975 from Western Sahara, and consisted of 37 males and 37 females (Cano 1988, Abáigar 1993).

Dama gazelle (*Nanger dama*) is a big desert/semidesert gazelle (body mass 40-75 Kg; Barbosa and Espeso 2005) sexually dimorphic in size. Adult males are 25% heavier than adult females. Males and females have horns, these being thicker and longer in males. Females are fertile at the age of 13-15 months (Alados and Escós 1991) and males about 16 (Espeso 2018). The gestation period is about 26 weeks. Female reproductive senescence is around 13-14 years. (Barbosa & Espeso 2005). Herds occur singly (males or females), or in mixed groups of 10 - 15, composed of a dominant adult male, several adult females, and young individuals. It is highly nomadic ranging widely in order to obtain sufficient nutrition. These gazelles used to undertake large seasonal migrations, moving north into the Sahara desert during the rainy season, and retreating south into the Sahel during the dry season. Founders of the captive population in Almería has been controversial. Following the criteria of "unknown parents", Cano (1991) considered that 16 individuals (3 males and 13 females) were the founders; coinciding with those animals included in the first published studbook of the species (Barbosa and Espeso 2005). However, Ruiz-López et al. (2009) using molecular information found that the founding

population of *N. dama mhorr* was much smaller. Finally, the history of its captive population at La Hoya was reconstructed based on information taken from Cano (1991) and references therein, Valverde (2004), and information available in the historical files of the Experimental Field Station “La Hoya” (Moreno et al. 2011). The current studbook derived from that reconstruction (Espeso 2018) assumes that five individuals (1 male and 4 females) should be considered as founders of the captive population of this gazelles at La Hoya, as many of the animals arriving at La Hoya from Western Sahara had been living in captivity for years and were actually relatives.

At La Hoya, once young adult males reach the age of 7-9 months, male-male interaction is common, and must be avoided to prevent injury. This social behaviour strongly determines the species management as enclosure must allow healthy animals to display the full range of normal behaviour (Escós 1992). Thus, at La Hoya animals are distributed in three different types of enclosures: a) breeding enclosure, generally occupied by a breeding group composed by one male and a group of 5 to 8 females their young (young males are removed when 7-9 months old to prevent injury by the adult male); b) bachelor enclosure, where young celibate males are kept forming bachelor groups; c) individual male enclosure where solitary males that have been used as breeders are kept (see Moreno & Espeso, 2008; López & Abáigar 2013, for detailed husbandry guidelines at La Hoya Field Station). Details on fecundity by age and sex, mortality and reproductive senescence are provided for gazelle species in Abáigar 2002, Barbosa & Espeso 2005, Moreno & Espeso 2008, López & Abáigar 2013).

Cited references

Abáigar, T., 2002. Conservación y manejo de una especie amenazada en cautividad. *Gazella dorcas neglecta* Registro Internacional. Madrid: Consejo Superior de Investigaciones Científicas (Biblioteca de Ciencias, 8).

Abáigar, T., 1993. Gazella dorcas neglecta Studbook. Instituto de Estudios Almerienses. Almería. ISBN: 84-8108-018-7.

Abáigar, T., Cano, M., 2005. Management and conservation of Cuvier’s gazelle (Gazella cuvieri) in captivity. International Studbook. Instituto de Estudios Almerienses. Almería. 102 pag.

Alados, C.L., 1982. Biología y Comportamiento de Gazella dorcas. Ph.D. Thesis, Universidad de Granada, Granada, Spain.

Alados, C.L., Escós, J., 1991. Phenotypic and genetic characteristics affecting lifetime reproductive success in female Cuvier's, dama and dorcas gazelles (*Gazella cuvieri, G. dama* and *G. dorcas*). Journal of Zoology 223: 307 – 321.

Barbosa, A., Espeso, G., 2005. International Studbook Gazella dama mhorr. CSIC. Madrid. ISBN: 84-00-08380-6.

Beudels, R.C., Devillers, P., Lafontaine, R.-M., Devillers-Terschuren, J., Beudels, M.-O., 2005. Sahelo-Saharan Antelopes. Status and Perspectives. CMS Technical Series Publication, Nº 11. UNEP/CMS Secretariat, Bonn. Germany.

Cano, M., 1991. El antílope Mohor, *Gazella (Nanger) dama mhorr* Bennett 1832 en cautividad. Granada: Universidad de Granada. Servicio de Publicaciones, D.L. 84-338-1334-X

Cano, M., 1988. Sobre las poblaciones de ungulados del Parque de Rescate de la Fauna Sahariana durante el período 1971-1986. Boletín del Instituto de Estudios Almerienses. Volumen Extraordinario: Homenaje a Manuel Cano Gea, pp: 281-292.

Cassinello, J., 1998. El arrui sahariano. Un caprino ancestral en Almería. Instituto de Estudios Almerienses. Almería.

Dickinson, T.G., Simpson, C.D., 1980. Home range, movements, and topographic selection of Barbary sheep in the Guadalupe Mountains, New Mexico. In Symposium on Ecology and Management of Barbary Sheep (C.D. Simpson, ed.) pp. 78-86. Lubbock: Texas Tech. Univ. Press.

Espeso, G., 2018. International Mhorr Gazelle Studbook, Nanger dama mhorr. Downloadable at http://www.eeza.csic.es/documentos/STBDA_18.txt

Gray, G.G., Simpson, C.D., 1982. Group dynamics of free-ranging Barbary sheep in Texas. Journal of Wildlife Management 46: 1096-1101.

Kingdon, J., 1997. The Kingdon Field Guide to African Mammals. Academic Press, Harcourt Brace & Co., New York, NY, USA.

López, L., Abáigar, T., 2013. Husbandry guidelines for captive breeding and management of saharawi dorcas gazelle (*Gazella dorcas neglecta*). Ed. CSIC, Madrid.

Moreno, E., Espeso, G., 2008. International studbook. Cuvier's gazelle (*Gazella cuvieri*). Ed. Ayuntamiento Roquetas de Mar-CSIC, Almería-Madrid.

Moreno, E., Ibáñez, M.B., Barbosa, A., 2011. Mother traits and offspring sex in two threatened gazelles species incaptivity. J. Nat. Conser. 19, 148-153.

Ruiz-López, M.J., Roldan, E.R.S., Espeso, G., Gomendio, M., 2009. Pedigrees and microsatellites among endangered ungulates: what do they tell us? Mol. Ecol. 18, 1352-1364.

Valverde, J.A., 2004. Sáhara, Guinea, Marruecos. Expediciones africanas. Memorias de un biólogo heterodoxo. Tomo III. Ed V&V, Madrid.

Appendix S2. Individuals filtered

Original pedigree files were downloaded from the studbooks available online at http://www.eeza.csic.es/es/programadecria.aspx, but not all individuals were made available for analysis. Several filtering criteria were introduced, aimed to avoid sources of bias for inbreeding and fitness estimates. Table S1 contains the original number of individuals in the studbooks, as well as the number of individuals removed in each filtering step, and the number of individuals retained in the processed pedigrees.

Table S1. Number of individuals during pedigree processing: Number of individuals in the studbook (N_{total}), number of individuals removed due to unknown ancestry (N_{UNK}), number of individuals removed as consequence of accidental death ($N_{accident}$), number of individuals at the end of the pedigree, removed for female productivity analysis (N_{recent} years), final number of individuals remaining for survival (N_{WS}) and productivity (N_{WP}) analysis.

Species	N_{total}	N_{UNK}	$N_{accident}$	N_{recent}	N_{WS}	N_{WP}
A. lervia	431	15	0	144	380	138
G. cuvieri	1778	696	5	297	948	388
G. dorcas	1845	19	1	484	1279	592
N. dama	2151	32	0	571	1316	611

First, individuals with unknown ancestry were removed. This includes individuals recorded in the studbooks with unknown (UNK) as well as “multiple” (MULT) ancestry, meaning that several individuals were candidate parents, but accurate ancestry could not be established. Descendants from these individuals were also removed. This filtering step was aimed to avoid downward biases of inbreeding that could affect analysis of inbreeding depression and purging. The higher number of individuals excluded for *G. cuvieri* compared to other species (N_{UNK} = 696) is mainly due to the higher degree of transference of this species to international institutions participating in its *Ex situ* Conservation Programme.

Then, individuals that died younger than 15 days of life as consequence of environmental accidents (e.g. trauma), were also removed, to avoid a downward bias of fitness due circumstances non-connected to genetics. This included individuals with identities 1057, 1381, 1384, 1386 and 1426 in *G. cuvieri*, and 1654 in *G. dorcas*. No individuals were removed for this reason for *A. lervia* and *N. dama*.

When considering lifetime female productivity as fitness (W_P in the main text), females at the end of the pedigree were also removed. The criteria was defined as a function of each species’ longevity, so that more long-lived species had more years of data removed from the pedigree. This is a required filter to accurately estimate inbreeding depression and purging for traits such as productivity, since in the more recent years, when individuals are more inbred, yet-alive individuals will have their productivity estimated underscored, and only complete records would be available for individuals that died earlier or died more prematurely. We selected the 90 % percentile of

longevity as threshold, and therefore removed the last 16 years of data for *A. lervia*, 11 years for *N. dama*, and 9 for the two *Gazella* species.

It must be noted that we only considered individuals belonging to La Hoya Experimental Field Station (see main text), and individuals from other locations had their fitness values set as NA (non available). Male productivity was always set to NA. As a last filtering step, individuals with NA values of fitness were also removed from the pedigrees, as long as they were not ancestors of individuals with non-NA values of fitness. Since this filtering is conditional to fitness, the number of individuals removed for survival and productivity analysis differs. The motivation of this last filtering step was to remove individuals not required for the remaining analyses, and to keep a shorter pedigree that improve the performance of downstream analyses.

Appendix S3. Complementary demographic parameters

The estimates of N_e reported in the main text were similar to those computed solving for N the classical non-overlapping generations expression $F = 1 - \left(1 - \frac{1}{2N}\right)^t$, where F is the average inbreeding for individuals in the last cohort and t is their average number of equivalent complete generations minus one, to account for the absence of self-fertilization: N_e = 3.87 (0.05), 14.10 (0.16), 39.58 (1.40) and 11.18 (0.11) for *A. lervia*, *G. cuvieri*, *G. dorcas* and *N. dama*, respectively (bootstrap errors parenthetically). This supports the approach proposed by Gutierrez et al. (2008, 2009), as well as the use of the classical expression with overlapping generations.

The effective number of ancestors (N_{ea}) defined as the minimum number of ancestors, founders or not, required to account for the IBD genetic diversity of the TP. The number of equivalent founder genomes (N_g), which also includes other sources of random loss of alleles such as genetic drift (Caballero and Toro 2000). Then, the existence of recent bottlenecks in the pedigree can be inferred when $\frac{N_{ea}}{N_{ef}} < 1$, and drift can be expected when $N_g < N_{ea}$. These parameters were estimated following the methods described by Boichard et al (1997). For the estimation of N_g, Monte-Carlo simulation was run for 10^4 iterations. They are reported in the table below. The effective number of equivalent founder genomes (N_g) approximates ½ N_f (N_f is the number of founders) for all species except for *G. dorcas*, for which it was lower (Figure S1).

Table S2. Effective number of ancestors N_{ae}, and number of equivalent founder genomes N_g (± SE).

**Species**	N_{ae}	N_g
A. lervia	1.77	1.03 (0.25)
G. cuvieri	3.58	2.00 (0.46)
G. dorcas	12.99	5.82 (1.01)
N. dama	2.61	1.87 (0.45)

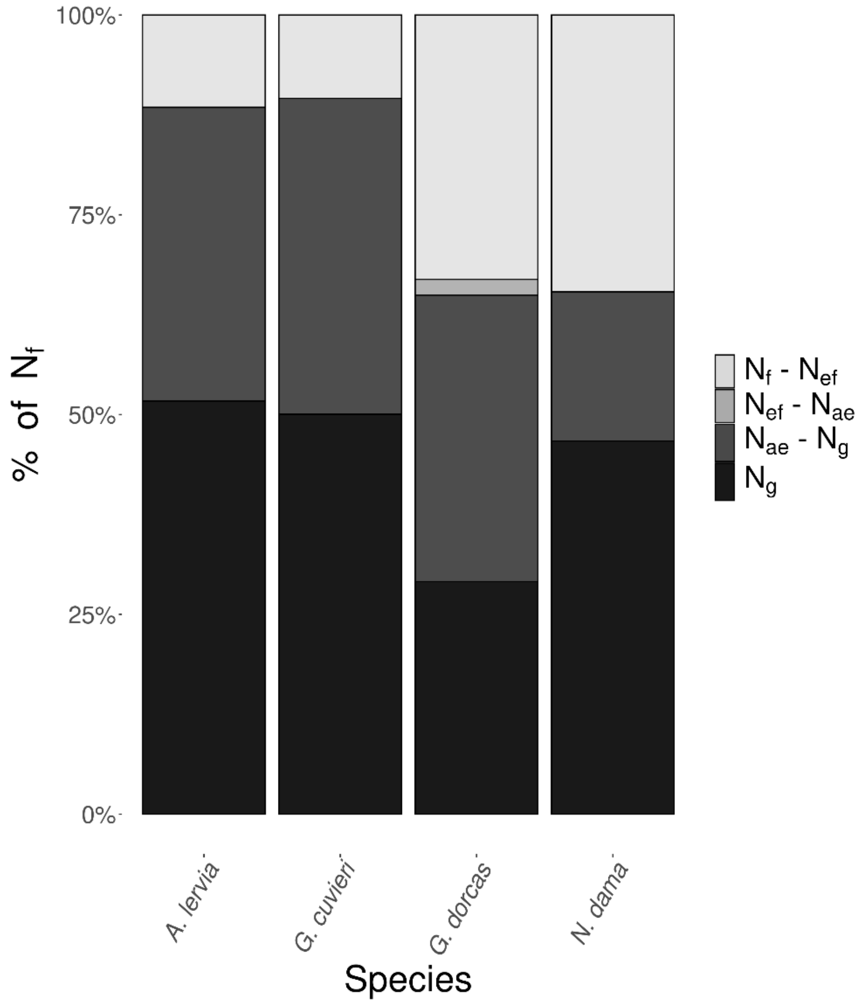

Figure S1. Partition of the number of founders (*Nf*) for different species (in %). The total height of the bar represents the total *Nf*. From top to bottom, stacked bars indicate the successive reduction of *Nf* after subtracting i) the component due to unbalanced founder contributions (*Nf-Nef*, in light grey), ii) the component due to unbalanced ancestor contributions (*Nef-Nae*, in grey), the component due to drift (*Nae-Ng*), and the number of equivalent founder genomes left (*Ng*).

Cited references

Boichard, D., Maignel, L., Verrier, E., 1997. The value of using probabilities of gene origin to measure genetic variability in a population. Genet. Sel. Evol. 29, 5-23.

Caballero, A., Toro, M., 2000. Interrelations between effective population size and other pedigree tools for the management of conserved populations. Genet. Res. 75, 331-343.

Appendix S3. Quantitative genetic analysis for survival

Genetic variability for the fitness traits was also evaluated in the full pedigree through the computation of the additive variance (h^2), using the R package MCMCglmm (Hadfield 2010), as in Gauzere et al. (2020). In the corresponding animal model, inbreeding and mother identity were included as potential random factors, and the most likely solution was selected using the deviance information criterion (DIC, Spiegelhalter et al. 2002). The dominance matrix was computed using the R package nadiv (Wolak 2012), and was also included in the model. Non-informative priors were chosen for all variables involved. MCMC was run for 3 x 10^5 iterations with a burn-in period of 5 x 10^4 iterations, and storing one in every 250 samples. Output were always check for low autocorrelation ($|r| < 0.1$), and effective sample sizes close to 10^3 for V_A. After running the animal model, V_A and h^2 were computed on the observed data scale using the QGglmm package (Villemereuil et al. 016). The mean-scaled additive variance (I_A) was also computed from the previous analyses by dividing the estimated V_A over the squared trait mean (Houle 1992, Hansen et al. 2011). An environment with R version 3.6.1 (R Core Team 2019) was used for all analyses.

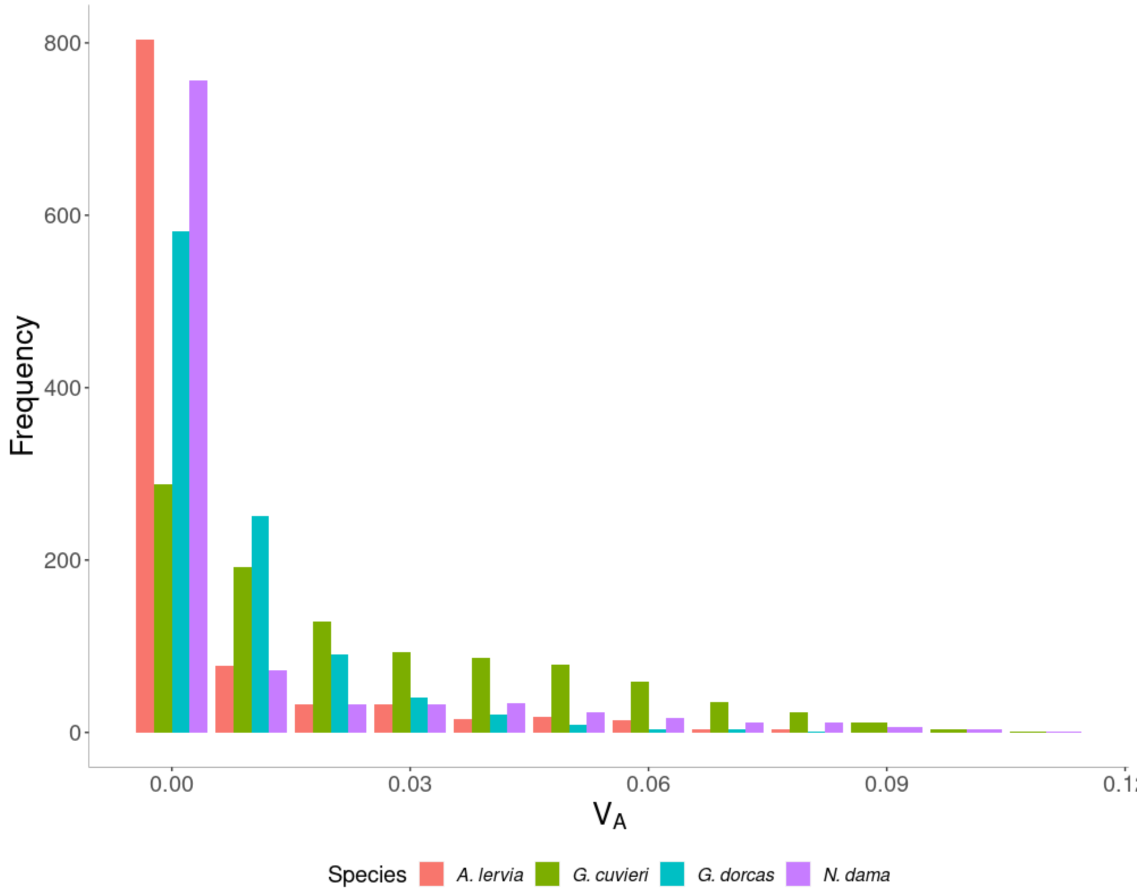

Figure S2. Posterior distribution of the additive variance (V_A) on 15-days survival. Frequency values of V_A based on 1000 MCMC samples are shown for *A. lervia* (red), *G. cuvieri* (green), *G. dorcas* (blue) and *N. dama* (purple). Note that V_A values shown have been multiplied by 100.

Cited references

de Villemereuil, P., Schielzeth, H., Nakagwa, S., Morrissey, M., 2016. General methods for evolutionary quantitative genetic inference from generalised mixed models. Genetics doi: 10.1534/genetics.115.186536.

Hadfield, J.D., 2010. MCMC Methods for multi-response generalized linear mixed model: The MCMCglmm R package. J. Stat. Softw. 33(2), 1-22.

Gauzere, J., Pemberton, J.M., Morris, S., Morris, A., Kruuk, L.E.B., Walling, C.A., 2020. The genetic architecture of maternal effects across ontogeny in the red deer. Evolution. doi:10.1111/evo.14000

Hansen TF, Pélabon C, Houle D. 2011. Heritability is not evolvability. Evol. Biol. 38: 258-277.

Houle D. 1992. Comparing evolvability and variability of quantitative traits. Genetics 130: 195-204.

R Core Team. 2019. R: A language and environment for statistical computing. R Foundation for Statistical Computing, Vienna, Austria. URL https://www.R-project.org/.

Spiegelhalter DJ, Best NG, Carlin BP, van der Linde A. 2002. Bayesian measures of model complexity and fit (with discussion). Journal of the royal statistical society, Series B 64(4): 583-639.

Wolak, ME. 2012. nadiv: an R package to create relatedness matrices for estimating non-additive genetic variances in animal models. Methods in Ecology and Evolution, 3(5): 792-796.

Table S3. PURGd results for the different models analyzed for *A. lervia*.

d coefficient	AICc	AICc-AICcMin	p-value H0 d=0	Δ	δ_M	b(sex:F)	b(sex:M)	b(yob)	b(POM:vet)	b(POM:init)
0.0773	321.994	0	0.34275436	0.261704	NA	NA	-0.058441	NA	0.127573	NA
0.18	322.452	0.458	0.23255268	0.235542	0.187701	NA	-0.0546754	NA	0.123292	NA
0.1395	322.575	0.581	0.29366557	0.169059	0.277558	NA	NA	NA	0.129155	NA
0.0439	323.157	1.163	0.32773472	0.294691	NA	NA	NA	NA	0.130879	NA
0.4561	324.908	2.914	0.01811814	0.381405	0.277702	NA	-0.0503571	NA	NA	NA
0.3618	325.335	3.341	0.02596182	0.654084	NA	NA	-0.0568096	NA	NA	NA
0.4435	325.59	3.596	0.01426111	0.431834	0.354186	NA	NA	NA	NA	NA
0.3518	327.1	5.106	0.00068851	0.587033	NA	NA	NA	4.00E-06	NA	NA
0.3716	328.694	6.7	0.01881617	0.549868	NA	NA	-0.024429	3.20E-05	NA	NA
0.2376	335.657	13.663	9.5434E-06	0.784905	-0.476827	NA	NA	2.60E-05	NA	NA
0.2649	360.771	38.777	1.8953E-10	1.56814	-1.5285	NA	-0.048712	-1.68E-13	NA	NA

Table S4. PURGd results for the different models analyzed for *G. cuvieri*

d coefficient	AICc	AICc-AICcMin	p-value H0 d=0	δ	δ_M	b(sex:F)	b(sex:M)	b(yob)	b(POM:vet)	b(POM:init)
0.4847	912.785	0	0.002134224	0.670733	0.660409	NA	-0.0969631	NA	NA	NA
0.4114	914.113	1.328	0.019905194	0.610545	0.62416	NA	-0.0936274	NA	0.0292959	NA
0.4639	917.957	5.172	0.038499876	0.630999	NA	NA	-0.0960652	NA	NA	NA
0.3478	918.153	5.368	0.139901704	0.568118	NA	NA	-0.0947323	NA	0.0266536	NA
0.4177	918.867	6.082	1.06305E-08	0.534397	0.354771	NA	-0.0909405	3.00E-06	NA	NA
0.4454	920.869	8.084	0.048016452	0.659681	NA	NA	-0.0824296	-2.00E-06	NA	NA
0.4886	921.135	8.35	0.001816458	0.723166	0.7288	NA	NA	NA	NA	NA
0.4131	921.421	8.636	0.017324388	0.648738	0.718073	NA	NA	NA	0.0363778	NA
0.4779	922.375	9.59	1.4544E-07	0.664942	0.52358	NA	NA	-1.68E-13	NA	NA
0.3717	926.171	13.386	0.126713715	0.595101	NA	NA	NA	NA	0.0261553	NA
0.4492	926.906	14.121	NA	0.689864	NA	NA	NA	5.00E-06	NA	NA

Table S5. PURGd results for the different models analyzed for *G. dorcas*

d coefficient	AICc	AICc-AICcMin	p-value H0 d=0	δ	δ_M	b(sex:F)	b(sex:M)	b(yob)	b(POM:vet)	b(POM:init)
0.4783	975.721	0	0.30818224	0.513157	0.246947	NA	-0.0573092	NA	0.108712	NA
0.4441	975.784	0.063	0.31157916	0.515606	NA	NA	-0.0589187	NA	0.10026	NA
0.4897	985.938	10.217	0.24618047	0.448798	-0.333022	NA	-0.0571574	NA	NA	NA
0.4901	986.72	10.999	0.19876713	0.437336	NA	NA	-0.0554997	NA	NA	NA
0.4878	1005.65	29.929	0.25432102	0.486644	0.360448	NA	NA	NA	0.108813	NA
0.4789	1006.39	30.669	0.29910058	0.492859	NA	NA	NA	NA	0.0956447	NA
0.4898	1015.88	40.159	0.23835951	0.427551	-0.20041	NA	NA	NA	NA	NA
0.4902	1015.97	40.249	0.24632628	0.422752	-0.188067	NA	NA	3.80E-06	NA	NA
0.4899	1016.22	40.499	4.8763E-11	0.422153	NA	NA	NA	-1.00E-06	NA	NA
0.49	994.219	18.498	NA	0.45862	-0.307137	NA	-0.0391415	1.60E-05	NA	NA
0.4899	991.356	15.635	NA	0.443079	NA	NA	-0.0446985	-1.69E-13	NA	NA

Table S6. PURGd results for the different models analyzed for *N. dama*

d coefficient	AICc	AICc-AICcMin	p-value H0 d=0000	δ	δM	b(sex:F)	b(sex:M)	b(yob)	b(POM:vet)	b(POM:init)
0.2302	1175.84	0	0.0278495	0.88246	0.356792	NA	-0.0481584	NA	NA	NA
0.2094	1176.57	0.73	0.04131397	1.10139	NA	NA	-0.0505622	NA	NA	NA
0.1964	1177.13	1.29	0.20642977	1.04263	NA	NA	-0.0499247	NA	0.0126292	NA
0.2254	1178.23	2.39	0.15984414	0.901784	0.308407	NA	-0.0429715	NA	0.0104916	NA
0.221	1179.82	3.98	0.02501352	0.976436	0.281283	NA	NA	NA	NA	NA
0.2449	1180.49	4.65	5.7686E-06	1.04679	NA	NA	-0.0250842	1.40E-05	NA	NA
0.2261	1180.51	4.67	0.15994035	0.986872	0.273232	NA	NA	NA	0.0036109	NA
0.1791	1180.72	4.88	0.18296803	1.08925	NA	NA	NA	NA	0.0151632	NA
0.2301	1181.16	5.32	1.412E-08	1.09049	NA	NA	NA	-1.69E-13	NA	NA
0.2595	1182.46	6.62	2.8513E-06	0.997569	0.141712	NA	NA	1.20E-05	NA	NA
0.2693	1183.12	7.28	9.9361E-10	1.02164	-0.0410967	NA	-0.0291742	1.60E-05	NA	NA

<u>Table S7.</u> Inbreeding-purging parameters estimated for productivity W_P: purging coefficient (*d*), P-value for the estimate of the purging coefficient, rate of inbreeding depression δ (i.e., estimated inbreeding load for *Wp* ascribed to the individual's genotype), maternal rate of inbreeding depression (i.e., estimate of the maternal inbreeding load δ_M , ascribed to the maternal component of the trait), sex effect (S, referred to males), period of management effect (*POM*, referred to the period when veterinary care becomes regular) and initial fitness (W_0, numerical estimate of the average fitness of the initial non-inbred population). NA values indicate that the factor was not selected in the model giving the best fit. The last column gives the average fitness for non-inbred individuals with non-inbred ancestors and its empirical standard error [$W_{0\ F=Fa=0}$ (±SE)].

Species	*d*	*P-value*	δ	δ_M	*POM*	W_0	$W_{0\ F=Fa=0}$
A. lervia	0.0	0.33	-2.25	-2.58	-0.59	11.8	10.50 (7.32)
G. cuvieri	0.0	0.32	-4.59	NA	-0.10	5.52	6.40 (1.91)
G. dorcas	0.0	0.32	-4.23	-0.08	-0.16	2.56	2.48 (0.34)
N. dama	0.0	0.32	-4.60	-1.09	NA	7.50	9.50 (3.47)

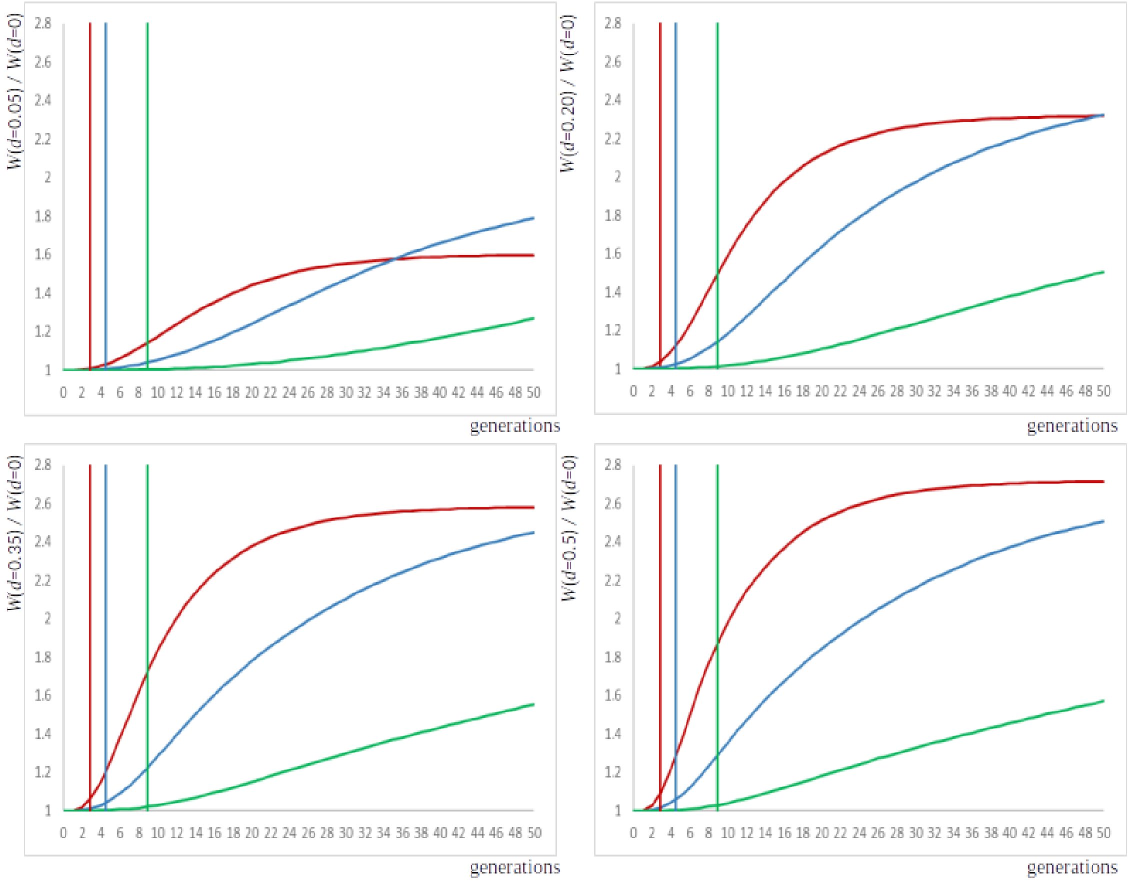

<u>Figure S3.</u> Evolution of the expected fitness *W* predicted under the Inbreeding-Purging model for a panmictic population (including random self-fertilization) using different purging coefficients (*d*=0.05, 0.2, 0.35 and 0.5) relative to the corresponding fitness expectation in the absence of purging (i.e., using *d*=0 to compute g_i), plotted against generation number for different effective population sizes: red, blue and green lines for N_e = 4, 10 and 40, respectively. Vertical lines correspond to the $t_m=\sqrt{(2N_e)}$ values with the same color code (rounded to the closest natural number).
